# Supplementary material for: Pol μ dGTP mismatch insertion opposite T coupled with ligation reveals promutagenic DNA repair intermediate
Source: Nat Commun. 2018 Oct 11;9:4213. doi: 10.1038/s41467-018-06700-5 (PMC6181931; doi:10.1038/s41467-018-06700-5)
Supplement: Supplementary file 1 — Supplementary Information [file 41467_2018_6700_MOESM1_ESM.pdf]

Supplementary Information

Pol µ dGTP mismatch insertion opposite T coupled with ligation reveals promutagenic DNA repair intermediate

Melike Çağlayan[#] and Samuel H. Wilson

Genome Integrity and Structural Biology Laboratory, National Institutes of Health, National Institute of Environmental Health Sciences, Research Triangle Park, NC 27709, USA

[#]Current address: Department of Biochemistry and Molecular Biology, University of Florida, Gainesville, FL 32610, USA

Correspondence: wilson5@niehs.nih.gov, caglayanm@ufl.edu

Supplementary Figures 1-11

Supplementary Table 1

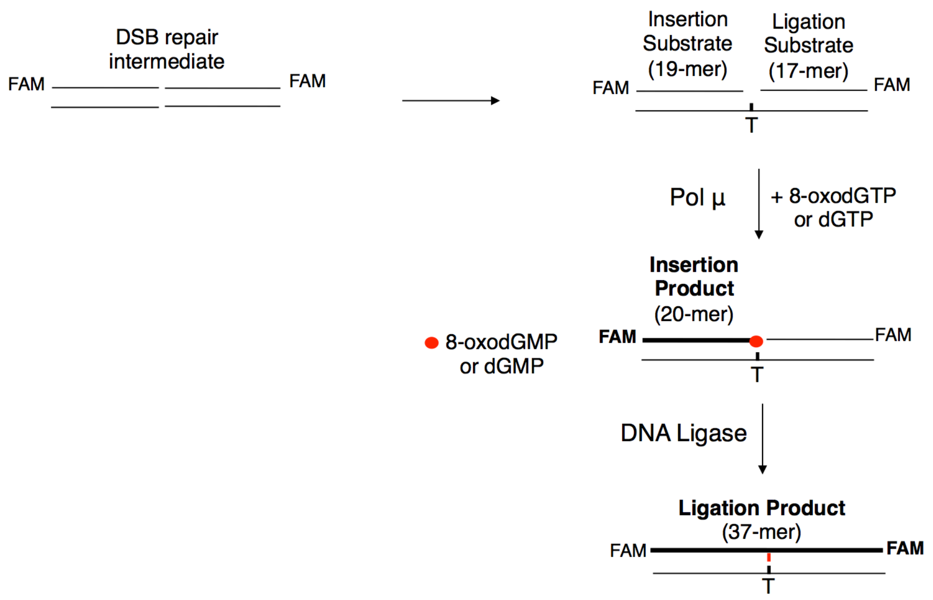

Supplementary Figure 1 Ilustration of a coupled DNA polymerase-ligase assay. The single-nucleotide gapped DNA substrate with template base T and the reaction products in the presence of pol μ, DNA ligase, and 8-oxodGTP or dGTP are indicated. FAM indicates the presence of a fluorescence tag.

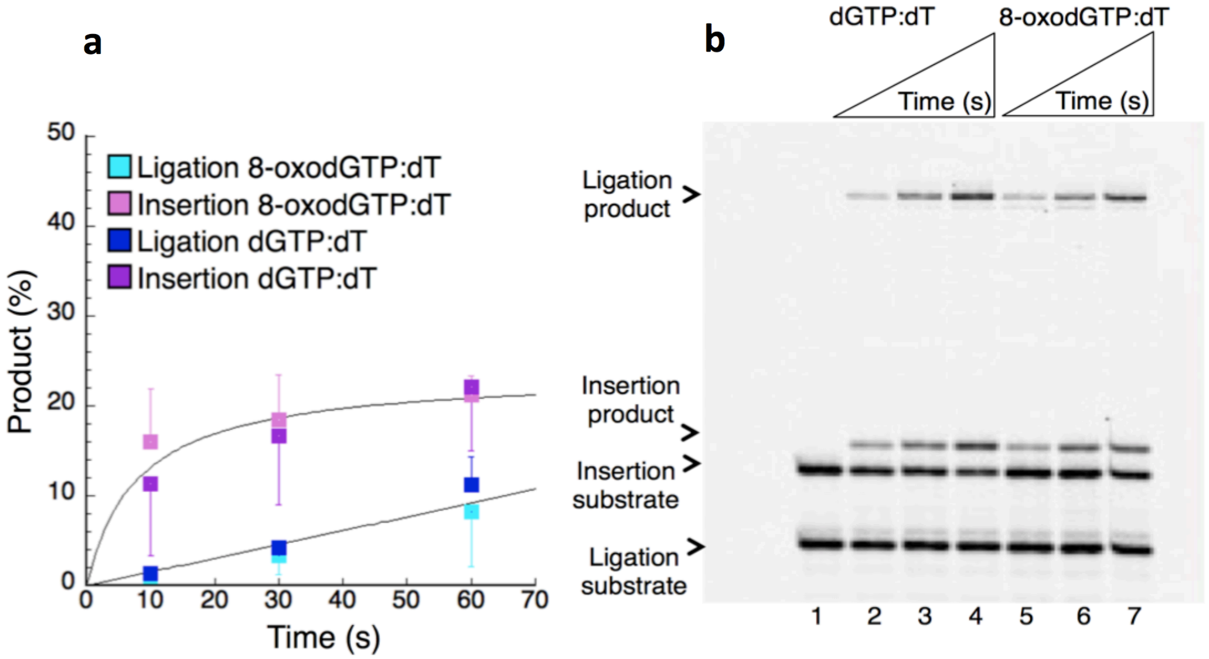

Supplementary Figure 2 Pol μ dGMP and 8-oxodGMP insertion coupled with ligation. (**a**) Graph shows time-dependent changes in the products of insertion and ligation. The data represent the average of four independent experiments ± SD, and are also presented in bar graph format (Fig. 1b). (**b**) Corresponding uncropped gel image presented in Fig. 1a. Lane 1 is the minus enzyme control for the single-nucleotide gapped DNA substrate with template base T. Lanes 2-4 and 5-7 are the reaction products in the presence of dGTP and 8-oxodGTP, respectively, and correspond to time points of 10, 30, and 60 s.

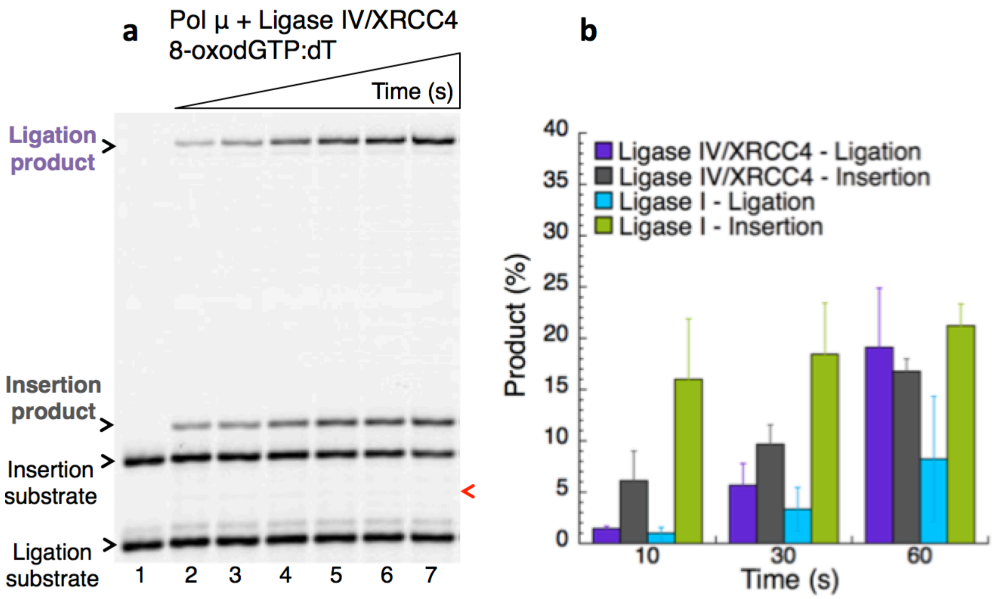

Supplementary Figure 3 Pol µ 8-oxodGMP insertion coupled with ligation by DNA ligase IV/XRCC4 complex. (**a**) Lane 1 is the minus enzyme control for the single-nucleotide gapped DNA substrate with template base T. Lanes 2-7 are the reaction products in the presence of 8-oxodGTP, and correspond to time points of 10, 20, 30, 40, 50, and 60 s, respectively. The position of the 5'-adenylate product is indicated by a red arrow. (**b**) Graph showing time-dependent changes and comparison between DNA ligase I vs DNA ligase IV/XRCC4 complex in the products of ligation and insertion. The data represent the average of three independent experiments ± SD

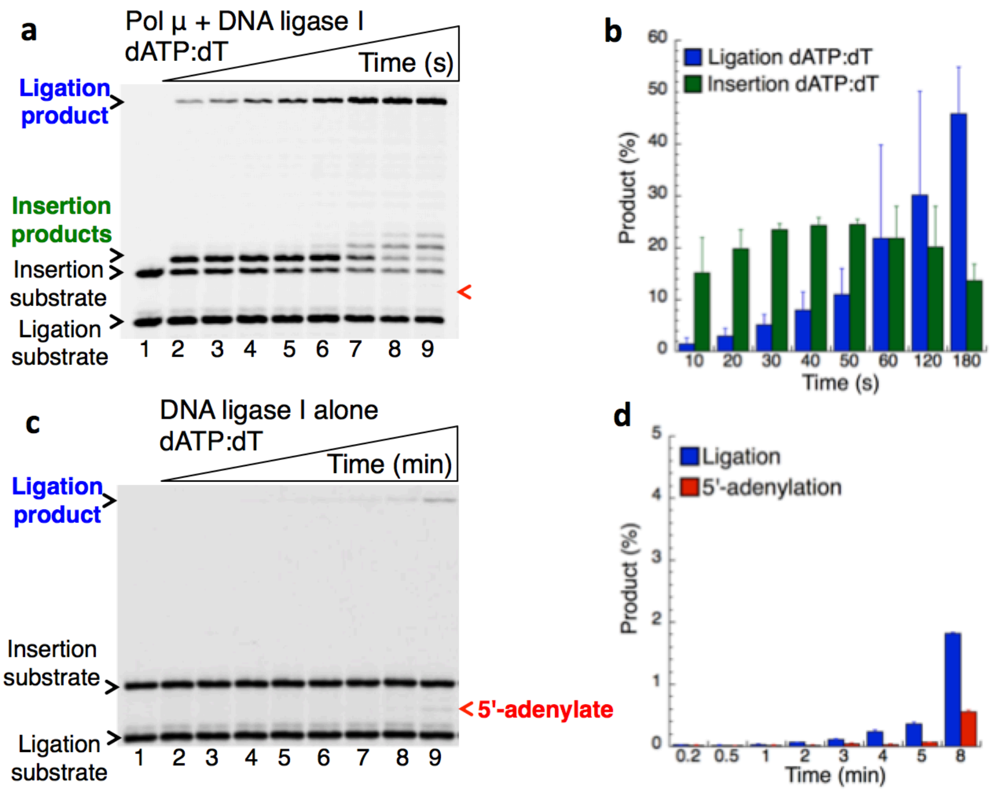

Supplementary Figure 4 (**a,b**) Pol μ dAMP insertion coupled with ligation. (**a**) Lane 1 is the minus enzyme control for the single-nucleotide gapped DNA substrate with template base T. Lanes 2-9 are the reaction products, in the presence of dATP, and correspond to time points of 10, 20, 30, 40, 50, 60, 120, and 180 s, respectively. (**b**) Graph showing time-dependent changes in the products of ligation and insertion. The data represent the average of three independent experiments ± SD. (**c,d**) Ligation of the single-nucleotide gapped DNA substrate by DNA ligase I itself. **c**, Lane 1 is the minus enzyme control for the single-nucleotide gapped DNA substrate with template base T. Lanes 2-9 are the reaction products, in the presence of dATP, and correspond to time points of 0.2, 0.5, 1, 2, 3, 4, 5, and 8 min, respectively. (**d**) Graph showing time-dependent changes in the products of ligation and 5'-adenylation (*i.e.*, ligation failure). The data represent the average of three independent experiments ± SD.

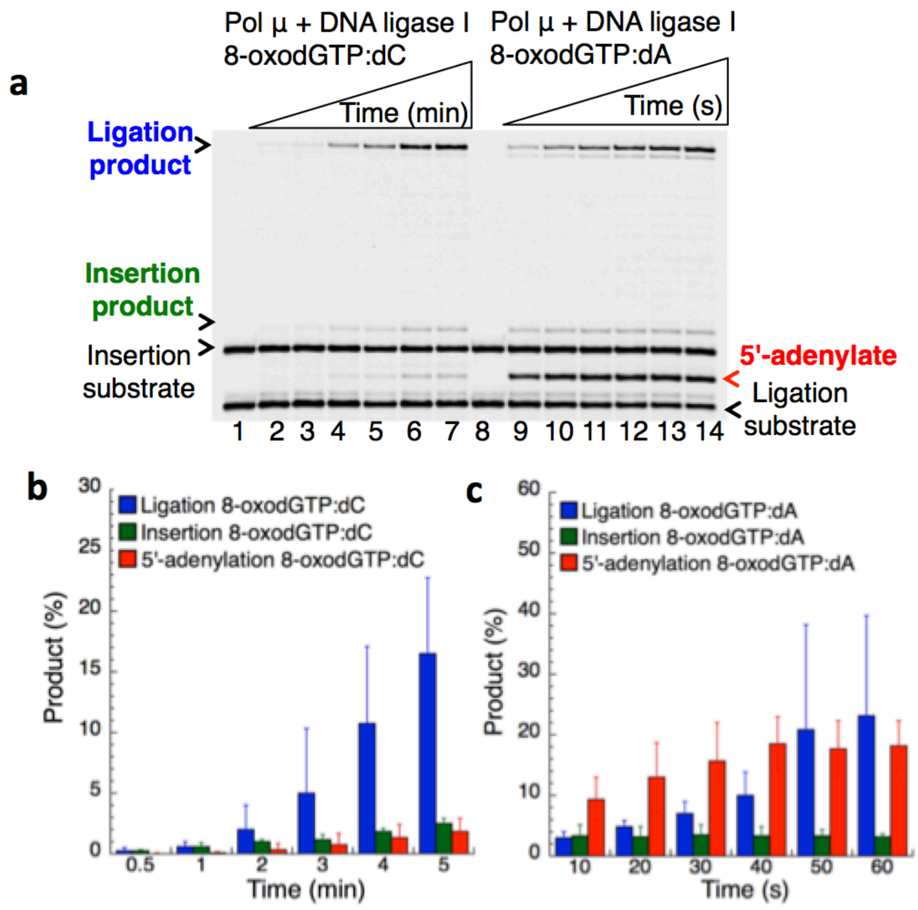

Supplementary Figure 5 Pol µ 8-oxodGTP:dC vs 8-oxodGTP:dA insertion coupled with ligation. (**a**) Lanes 1 and 8 are the minus enzyme controls for the single-nucleotide gapped DNA substrate with template base C or A, respectively. Lanes 2-7 are the reaction products for pol µ 8-oxodGTP:dC and correspond to time points of 0.5, 1, 2, 3, 4, and 5 min, respectively. Lanes 9-14 are the reaction products for pol µ 8-oxodGTP:dA and correspond to time points of 10, 20, 30, 40, 50, and 60 s, respectively. Graphs showing time-dependent changes in the products for pol µ 8-oxodGTP:dC (**b**) and pol µ 8-oxodGTP:dA (**c**). The data represent the average of three independent experiments ± SD.

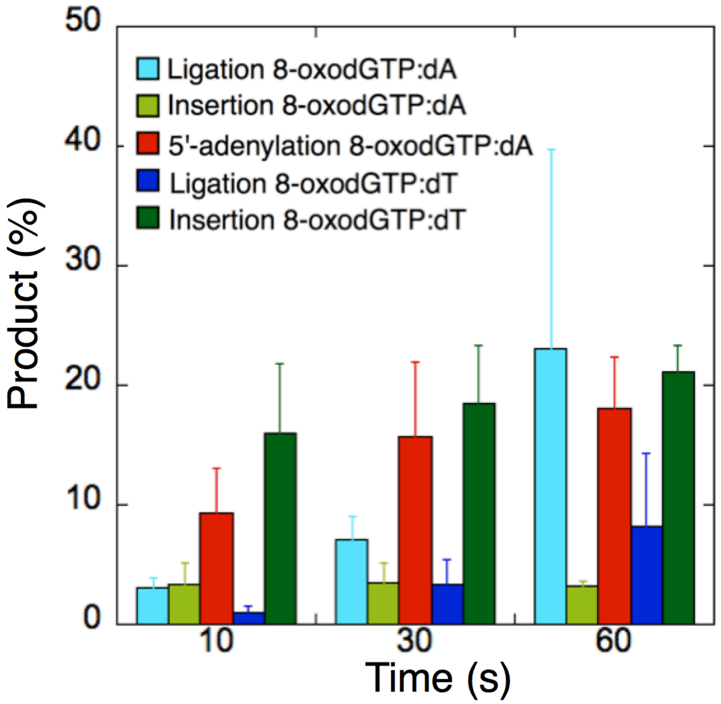

Supplementary Figure 6 The comparision of pol μ 8-oxodGTP:dA vs 8-oxodGTP:dT insertion coupled with ligation. Graphs showing time-dependent changes in the products for 8-oxodGTP:dT and 8-oxodGTP:dT were presented in Fig. 1b and Supplementary Fig. 4c, respectively. The data represent the average of three independent experiments ± SD.

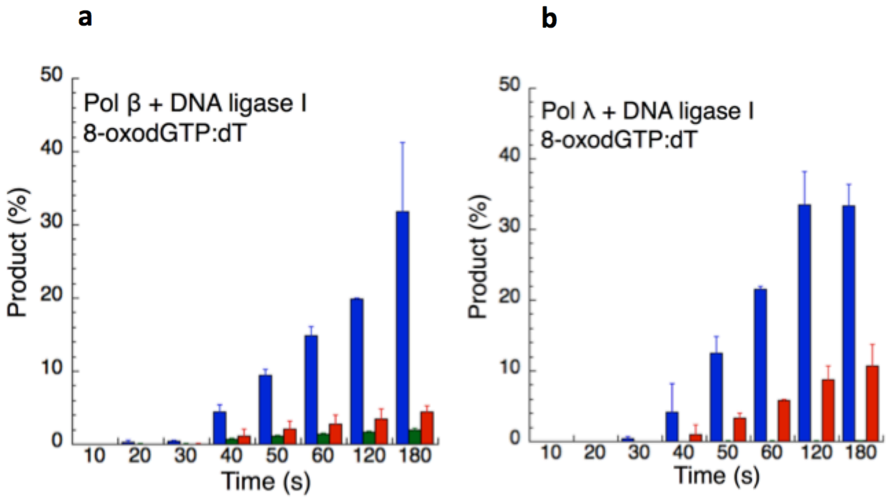

Supplementary Figure 7 Pol β vs Pol λ 8-oxodGTP:dT insertion coupled with ligation. Graphs showing time-dependent changes in the products of ligation (blue) and 5'-adenylation (red) for pol β 8-oxodGTP:dT (**a**) and pol λ 8-oxodGTP:dT (**b**). The data represent the average of three independent experiments ± SD. The data for the ligation coupled with pol β 8-oxodGTP:dT was reported[11]; in this study, we repeated the experiments for comparison with pol λ and pol μ.

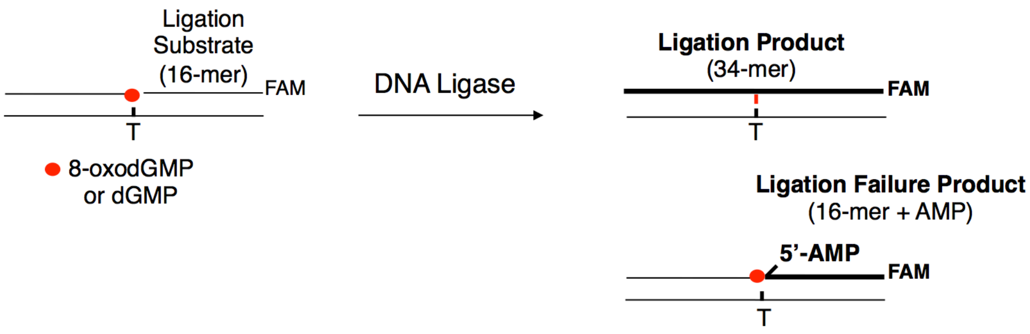

Supplementary Figure 8 Ilustration of ligation assay. The nicked DNA substrate with 3'-preinserted 8oxodG or dG opposite template base T; the reaction products in the presence of DNA ligase alone are indicated. FAM indicates the presence of a fluorescence tag.

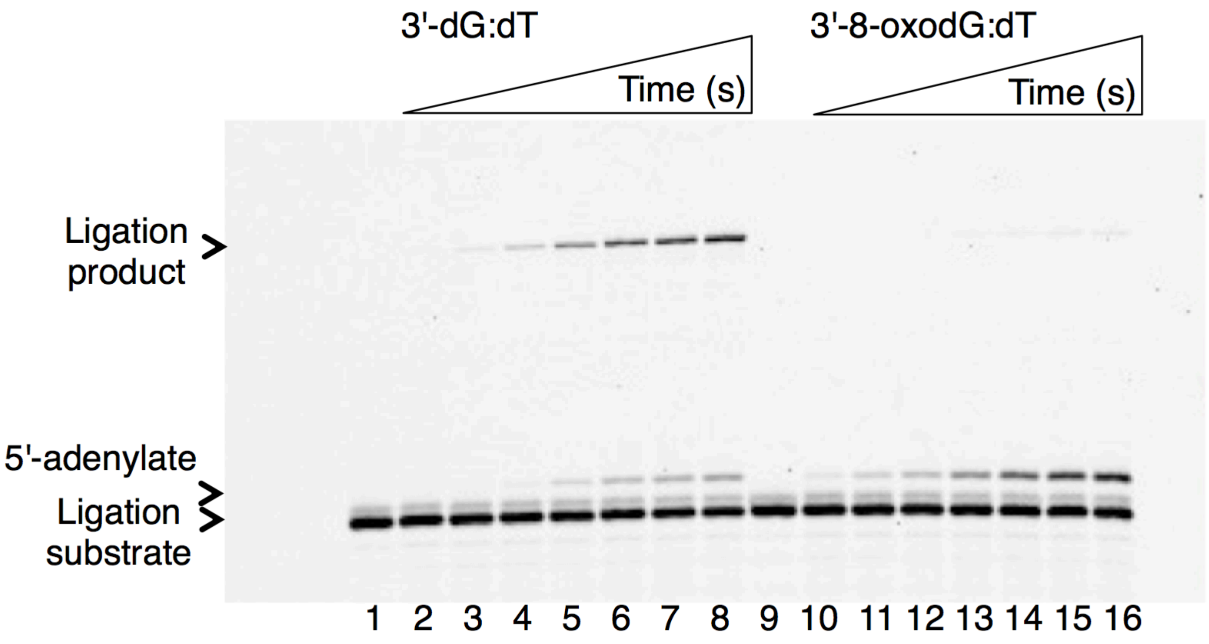

Supplementary Figure 9 The uncropped gel image for the ligation of 3'-preinserted dG and 8-oxodG (Fig. 2). Lanes 1 and 9 are the minus enzyme controls for the nicked DNA substrate with 3'-8-oxodG and 3'-dG, respectively. Lanes 2-8 and 10-16 are the reaction products, and correspond to time points of 10, 30, 60, 120, 180, 240, and 300 s.

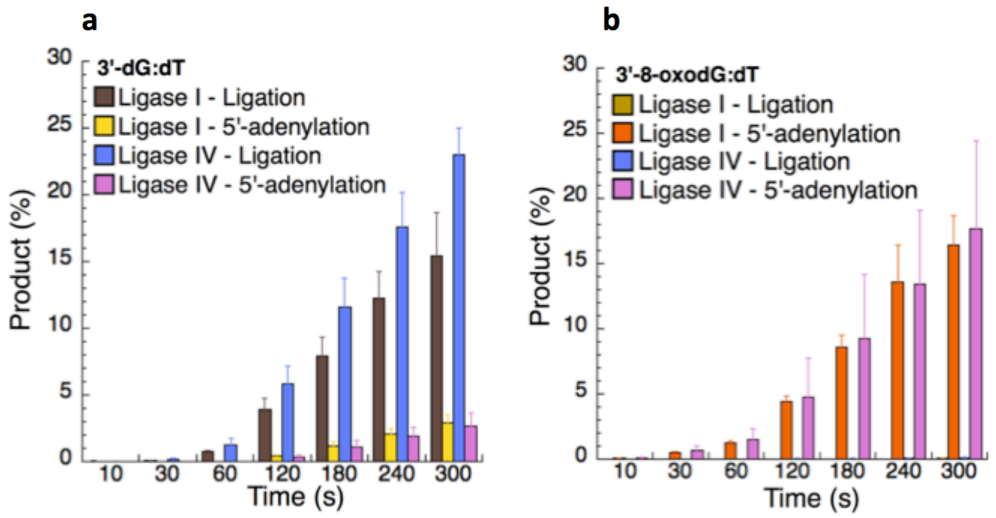

Supplementary Figure 10 Comparison of ligation by DNA ligase I vs Ligase IV in the presence of 3'-mismatched ends. Graphs showing time-dependent changes in the products of ligation and 5'-adenylation for 3'-dG:dT (**a**) and 3'-8-oxodG:dT (**b**) preinserted mismatches. The data represent the average of three independent experiments ± SD.

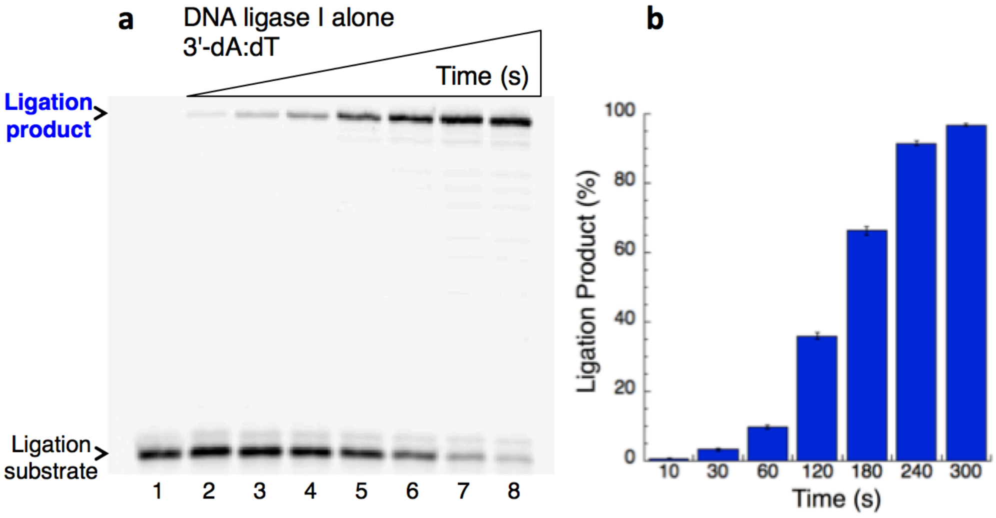

Supplementary Figure 11 Ligation of 3'-preinserted correct dA:dT base pair. (**a**) Lane 1 is the minus enzyme control for the nicked DNA substrate with template base dT. Lanes 2-8 are the ligation products and correspond to time points of 10, 30, 60, 120, 180, 240, and 300 s, respectively. (**b**) Graph showing time-dependent changes in the products of ligation. The data represent the average of three independent experiments ± SD.

Oligonucleotide	Substrate	Sequence (5' to 3')
1	Upstream Primer (19-mer)	FAM-AACATGGGCGGCATGAAAT
2	Downstream Primer (17-mer)	AATGCCCATCCTCACCA-FAM
3	Template A (37-mer)	TGGTGAGGATGGGCATTAATTTCATGCCGCCCATGTT
4	Template C (37-mer)	TGGTGAGGATGGGCATTCATTTCATGCCGCCCATGTT
5	Template T (37-mer)	TGGTGAGGATGGGCATTTATTTCATGCCGCCCATGTT
6	Upstream Primer/8-oxoG (18-mer)	CATGGGCGGCATGAACCX
7	Upstream Primer/dG (18-mer)	CATGGGCGGCATGAACCG
8	Upstream Primer/dA (18-mer)	CATGGGCGGCATGAACCA
9	Downstream Primer (16-mer)	GAGGCCCATCCTCACC
10	Template T (34-mer)	GGTGAGGATGGGCCTCTGGTTCATGCCGCCCATG

Supplementary Table 1 DNA substrates used in this study. Oligonucleotides 1-5 are used to construct the single-nucleotide gapped DNA substrates with template bases A, C, or T. Oligonucleotides 6-10 are used to construct the nicked DNA substrates with 3'-preinserted 8oxodG, dG, or dA opposite template base T. FAM indicates the presence of a fluorescence tag.
